# Genetic Polymorphisms Associated to Folate Transport as Predictors of Increased Risk for Acute Lymphoblastic Leukemia in Mexican Children

**DOI:** 10.3389/fphar.2016.00238

**Published:** 2016-08-05

**Authors:** Fausto Zaruma-Torres, Ismael Lares-Asseff, Aurea Lima, Aarón Reyes-Espinoza, Verónica Loera-Castañeda, Martha Sosa-Macías, Carlos Galaviz-Hernández, María C. Arias-Peláez, Miguel A. Reyes-López, Luis A. Quiñones

**Affiliations:** ^1^Pharmacogenomics Academia, National Polytechnic Institute-CIIDIRDurango, Mexico; ^2^School of Biochemistry and Pharmacy, University of CuencaCuenca, Ecuador; ^3^CESPU, Instituto de Investigação e Formação Avançada em Ciências e Tecnologias da SaúdeGandra, Portugal; ^4^State Center of Cancerology of SSADurango, Mexico; ^5^Institute of Scientific Research of the University Juarez of State of DurangoDurango, Mexico; ^6^Center of Biotechnology Genomics, National Polytechnic InstituteReynosa, Mexico; ^7^Pharmacological and Molecular Program, Laboratory of Chemical Carcinogenesis and Pharmacogenetics (CQF), Faculty of Medicine, ICBM, University of ChileSantiago, Chile

**Keywords:** acute lymphoblastic leukemia, folate transporters, genetic polymorphisms, methotrexate, molecular epidemiology

## Abstract

Acute lymphoblastic leukemia (ALL) is a frequent neoplasia occurring in children. The most commonly used drug for the treatment of ALL is methotrexate (MTX), an anti-folate agent. Previous studies suggest that folate transporters play a role in ALL prognosis and that genetic polymorphism of genes encoding folate transporters may increase the risk of ALL. Therefore, the main goal of this study was to determine the associations among six genetic polymorphisms in four genes related with the folate transporter pathway to determine a relationship with the occurrence of ALL in Mexican children. A case-control study was performed in 73 ALL children and 133 healthy children from Northern and Northwestern Mexico. *COL18A1* (rs2274808), *SLC19A1* (rs2838956), *ABCB1* (rs1045642 and rs1128503), and *ABCC5* (rs9838667 and rs3792585). Polymorphisms were assayed through qPCR. Our results showed an increased ALL risk in children carrying CT genotype (OR = 2.55, CI 95% 1.11–5.83, *p* = 0.0001) and TT genotype (OR = 21.05, CI 95% 5.62–78.87, *p* < 0.0001) of *COL18A1* rs2274808; in *SLC19A1* rs2838956 AG carriers (OR = 44.69, CI 95% 10.42–191.63, *p* = 0.0001); in *ABCB1* rs1045642 TT carriers (OR = 13.76, CI 95% 5.94–31.88, *p* = 0.0001); in *ABCC5* rs9838667 AC carriers (OR = 2.61, CI 95% 1.05–6.48, p < 0.05); and in *ABCC5* rs3792585 CC carriers (OR = 9.99, CI 95% 3.19–31.28, *p* = 0.004). Moreover, several combinations of genetic polymorphisms were found to be significantly associated with a risk for ALL. Finally, two combinations of *ABCC5* polymorphisms resulted in protection from this neoplasia. In conclusion, certain genetic polymorphisms related to the folate transport pathway, particularly *COL18A1* rs2274808*, SLC19A1* rs2838956*, ABCB1* rs1045642, and *ABCC5* rs3792585, were associated with an increased risk for ALL in Mexican children.

## Introduction

Acute lymphoblastic leukemia (ALL) is a malignant cancer disorder with an etiology not yet completely understood (Zeller et al., 2005). Its prevalence of ~34 to 35:100,000 in the Mexican pediatric population, accounting for 80–85% of all childhood leukemia found in northwestern Mexico (Rodríguez et al., 2010). Many factors, such as, physical, chemical, and genetic causes, are associated with ALL susceptibility in pediatric patients (Skibola et al., 1999).

The choice of chemotherapy treatment for ALL is based on the St. Jude Total XV protocol with antifolate drugs, as methotrexate (MTX; Pui et al., 2009, 2010). Three cellular mechanisms for folate transport have been identified: folate receptors (FR), reduced folate carrier (RFC), and the newly described proton-coupled folate transporter (PCFT). RFC 1 (reduced folate carrier 1), a 57–65 kDa integral transmembrane and energy-dependent protein, also called SLC19A1, member of the SLC19 family of solute carriers belonging to the ABC (ATP-binding cassette transporter family; Takatori et al., 2006) is the primarily way for folate or antifolate drugs transport (Sirotnak and Tolner, 1999; Ganapathy et al., 2004).

RFC1 is encoded by *RFC1* (*SLC19A1)* gene located at chromosome 21 (*locus* 21q22.2-q22.3; Moscow et al., 1997; Jansen and Pieters, 1998). Moscow et al. demonstrated that *RFC1* is over-expressed in cancer cell lines, mainly in breast cancer and leukemia. This over-expression produces an increased *in vitro* cytotoxicity due to contact with MTX, which may explain the affinity of these receptors to the anti-folates (Moscow et al., 1997). RFC1 transport function is enhanced by another protein, Collagen alpha-1 (XVIII) chain, encoded by *COL18A1* (*locus* 21q22.3), and has been described as crucial for malignant processes due to endostatin production (Digtyar et al., 2007), which is a powerful angiogenesis and tumor growth inhibitor (Sertie et al., 2000).

Conversely, MDR1 and MRP5, belonging to an important efflux transporter protein family of drugs and their metabolites called multidrug resistance proteins (MDR and/or MRP), have been shown to be important in the treatment of cancer cells (Higgins, 2001; Holland and Holland, 2005). These proteins are encoded by highly polymorphic genes (*ABCB1* and *ABCC5*) that have been associated with increased ALL risk and have also been implicated in oncologic treatment interpatient variability (Gottesman et al., 2002; Brisson et al., 2015), specifically against anti-folate drugs (Wielinga et al., 2005), leading to an increased risk of relapse (Choi, 2005). There is evidence that MRP5 transporter is over-expressed in the biological barriers of the brain, which could support the idea that ALL patients frequently relapse in the central nervous system (Warren et al., 2009); therefore, some genotypic modifications in the gene *ABCC5* would enhance the severity of ALL. Disruptions of the homeostasis of the one-carbon metabolism are attributed to folate deficiencies, leading to DNA damage. Therefore, genetic defects and polymorphisms in these pathways may have influence in cancer susceptibility and therapeutic response (Homberger et al., 2000).

Therefore, in this research we aimed to evaluate the influence of six genetic polymorphisms in these membrane folate transport associated-proteins on the ALL susceptibility development, to expand the understanding of these variants as potential genetic risk factors for ALL pathogenesis.

## Materials and methods

### General study design

This research was performed as an observational prospective, case-control, association study. This study was approved on April 17th, 2013 by the CECAN Ethics and Research Committee, Durango, Mexico, in accordance with the Helsinki Declaration, Good Clinical Practices (CGP), and Mexican General Health Law. Signed informed assent and consent were obtained from all children, and patients and controls were tutored before participation in the study.

Seventy-three pediatric ALL patients were admitted between May 2013 and December 2014 to the Hematology-Oncology Unit, Durango State Cancer Center (Centro Estatal de Cancerología, CECAN), Durango, Mexico. The ALL diagnosis was based on the Franco-American British Association criteria (Lilleyman et al., 1986). One hundred and thirty-three children without ALL were used as the control group.

### Genotyping

DNA extraction was performed using total blood samples obtained by venous punctures using a commercial kit (Macherey-Nagel®, Düren, Germany). After extraction, DNA integrity and purity were evaluated by horizontal electrophoresis in a 1% agarose gel stained with ethidium bromide. The concentration and quality were analyzed in a NanoDrop 2000® (Thermo Scientific, Wilmington, DE, USA). Determination of single nucleotide polymorphisms (SNPs) was analyzed using real-time polymerase chain reaction (qPCR) by 48-well plate StepOne® Real-Time PCR system (Applied Biosystems, Carlsbad, CA, USA) with *TaqMan* probes by Applied Biosystems StepOne™ (Foster City, CA, USA). The SNPs *COL181* (rs2274808)*, SLC19A1* (rs2838956), *ABCB1* (rs1045642, rs1128503), and *ABCC5* (rs9838667, rs3792585) were determined by a typical reaction containing 5 ng/μl of DNA, 0.5 μL of 20X *TaqMan* SNP genotyping assay and 5.0 μL of 2X *TaqMan* Genotyping Master Mix (Foster City, CA, USA).

### Statistical analyses

Hardy-Weinberg equilibrium (HWE) and binding disequilibrium analyses were conducted using expected and observed genotypic and allelic frequencies in the study population. The SNPStats (2006, Catalan Institute of Oncology, Barcelona, Spain) software was used (Solé et al., 2006).

The relative risk associations of the *COL18A1, ABCB1*, and *ABCC5* genetic polymorphisms with ALL susceptibility were assessed and were expressed as an odds ratio (OR) or a *p* < 0.05 with a 95% confidence interval (CI 95%). In addition, analyses of the associations between binary combinations of polymorphisms within the same *locus* and ALL were made to establish the relationship with the pathology. Finally, we developed an artificial neural network architecture of three layers. The first layer with covariates and factors, the second and hidden layer was from 60 to 2 neurons, and the third layer was the presence or absence of ALL (defined as the dependent variable). SAS v9.0 (USA, 2002) and Statistica v7 (USA, 2004) softwares were used. Fifty subjects were used for the training phase. The best model was obtained by comparison through the 2log Likelihood criteria and with the lower relative classification error.

## Results

Pediatric ALL patients and the controls showed a median age of 7.92 and 5.85 years, respectively. The anthropometric characteristics and biological parameters are presented in Table 1. As expected, a difference in the pathognomonic variables was observed between the groups.

**Table 1:** **Anthropometric characteristics and biochemical parameters of subjects**.

**Variable**	**Case *N* = 73 (median, range)**	**Control *N* = 133 (median, range)**	*p*-value^*^
Age(years)	7.92 (1.15–18.08)	5.85 (1.43–16.13)	0.022
Gender (male/female)	40 (54.79)/33 (45.21)	62 (47%)/71 (53%)	0.612
Body Weight (Kg)	17.5 (11.4–55.1)	22.9 (15.3–53.7)	0.120
Height (m)	1.07 (0.65–1.71)	1.22 (0.67–1.63)	0.216
Body surface (m^2^)	0.67 (0.43–1.7)	0.77 (0.57–1.34)	0.450
BMI (Kg/m^2^)	16.65 (12.36–28.1)	21 (18.5–27.5)	0.030
Time of treatment (weeks)	76 (25–289)	–	–
Dose MTX (low-high)	40.1 mg/m^2^–5 g/m^2^	–	–
Hemoglobin (g/dL)	11.97 (7.00–16.8)	12.55 (11.06–14.3)	0.876
Hematocrit (%)	35.45 (27.2–47.0)	36.6 (34.6–45.1)	0.667
Erythrocyte (cell/mm^3^)	4.15 (2.85–5.89) × 10^6^	4.3 (4.0–6.54) × 10^6^	0.890
Leukocyte (cell/mm^3^)	3.61 (1.079–17.6) × 10^3^	7.06 (5.9–14.2) × 10^3^	0.011
Platelet (cell/mm^3^)	185.2 (22.2–378) × 10^3^	255 (241–790) × 10^3^	0.003
AST (UI/L)	36.85 (0.4–149)	14.0 (9.2–39.5)	0.010
ALT (UI/L)	46.6 (2.3–210)	19.4 (1.9–69)	0.006
Uric Acid (mg/dL)	3.42 (2.05–4.77)	3.77 (3.1–4.86)	0.758
Urea(mg/dL)	11.49 (8.89–61.75)	34.2 (10.7–63.2)	0.035
Creatinine (mg/dL)	0.70 (0.24–1.54)	0.8 (0.42–1.32)	0.870
Alkaline phosphatase (UI/L)	257 (46–359)	62 (42–335)	0.001

* p < 0.05.

Table 2 shows the allelic and genotypic frequencies for the study groups and HWE values for the control group. The results indicated five evaluated SNPs were in HWE, except *ABCB1* (rs1128503). In addition, we determined significant differences in allelic frequencies between SNPs.

**Table 2:** **Allele and genotype frequencies of *COL18A1, SLC19A1, ABCB1, ABCC5* polymorphisms in subjects**.

***Gene***	**SNP**		***Genotype***				**Allele**		
			**Case n (%)**	**Control n (%)**	HWE *p*-value^*^		**Case n (%)**	**Control n (%)**	**χ^2^**
*COL18A1*	rs2274808	*CC*	39 (0.53)	107 (0.80)	0.205	*C*	93 (0.64)	237 (0.89)	36.57^*^
		*CT*	15 (0.21)	23 (0.17)		*T*	53 (0.36)	29 (0.11)	
		*TT*	19 (0.16)	6 (0.02)					
*SLC19A1*	rs2838956	*AA*	0 (0.00)	48 (0.36)	0.42	*A*	75 (0.51)	156 (0.59)	1.74
		*AG*	71 (0.97)	60 (0.45)		*G*	71 (0.49)	110 (0.41)	
		*GG*	2 (0.03)	25 (0.19)					
*ABCB1*	rs1045642	*CC*	35 (0.48)	70 (0.53)	0.541	*C*	93 (0.64)	191 (0.72)	2.53
		*CT*	23 (0.32)	51 (0.38)		*T*	53 (0.36)	75 (0.28)	
		*TT*	15 (0.21)	12 (0.09)					
	rs1128503	*CC*	35 (0.48)	76 (0.57)	0.09	*C*	99 (0.68)	196 (0.74)	1.32
		*CT*	29 (0.40)	44 (0.33)		*T*	47 (0.32)	70 (0.26)	
		*TT*	9 (0.12)	13 (0.10)					
*ABCC5*	rs9838667	*AA*	47 (0.64)	88 (0.66)	0.139	*A*	109 (0.75)	213 (0.80)	1.36
		*AC*	15 (0.21)	37 (0.28)		*C*	37 (0.25)	53 (0.20)	
		*CC*	11 (0.15)	8 (0.06)					
	rs3792585	*TT*	18 (0.25)	93 (0.70)	0.541	*T*	145 (0.74)	222 (0.83)	6.21^*^
		*TC*	20 (0.27)	36 (0.27)		*C*	51 (0.26)	44 (0.17)	
		*CC*	35 (0.48)	4 (0.03)					

* p < 0.05.

To analyze the binary association responses, five inheritance models were estimated (codominant, dominant, over-dominant, recessive, and log-additive; Solé et al., 2006; Table 3). The best model estimation was determined as a function of lower values using the Akaike Information Criterion (AIC) and Bayesian Information Criterion (BIC) compared to a reference model (codominant: *XX*, wild type; *XY*, heterozygous; *YY*, homozygous) Finally, the association with risk for ALL was estimated.

**Table 3:** **Individual risk analyses for SNPs in relation to ALL**.

**Gene (SNP)**	**Model**	**Genotype**	**Case n (%)**	**Control n (%)**	**OR**	**95%CI**	*p*-value^*^	**AIC**	**BIC**
***COL18A1*** **(rs2274808)**									
Codominant		*CC*	39 (53.4%)	107 (80.5%)	1.00	Reference	0.0001	240.0	260.0
		*CT*	15 (20.6%)	23 (17.3%)	2.55	(1.11–5.83)			
		*TT*	19 (26%)	3 (2.3%)	21.05	(5.62–78.87)			
Dominant		*CC*	39 (53.4%)	107 (80.5%)	1.00	Reference	< 0.0001	248.6	265.2
		*CT-TT*	34 (46.6%)	26 (19.6%)	5.01	(2.48–10.13)			
***SLC19A1*** **(rs2838956)**									
Codominant		*AA*	0 (0%)	48 (36.1%)	1.00	Reference	< 0.0001	200.5	220.5
		*AG*	71 (97.3%)	60 (45.1%)	0.00	NA			
		*GG*	2 (2.7%)	25 (18.8%)	0.00	NA			
Overdominant		*AA-GG*	2 (2.7%)	73 (54.9%)	1.00	Reference	< 0.0001	202.9	219.6
		*AG*	71 (97.3%)	60 (45.1%)	44.69	(10.42–191.63)			
***ABCB1*** **(rs1045642)**									
Codominant		*CC*	15 (20.6%)	70 (52.6%)	1.00	Reference	< 0.0001	223.6	243.6
		*CT*	23 (31.5%)	51 (38.4%)	1.67	(0.77–3.63)			
		*TT*	35 (48%)	12 (9.0%)	17.43	(6.91–43.97)			
Recessive		*CC-CT*	38 (52%)	121 (91.0%)	1.00	Reference	< 0.0001	223.3	239.9
		*TT*	35 (48%)	12 (9.0%)	13.76	(5.94–31.88)			
***ABCB1*** **(rs1128503)**									
Codominant		*CC*	35 (48%)	76 (57.1%)	1.00	Reference	0.68	271.7	291.7
		*CT*	29 (39.7%)	44 (33.1%)	1.33	(0.71–2.50)			
		*TT*	9 (12.3%)	13 (9.8%)	1.19	(0.45–3.15)			
Overdominant		*CC-TT*	44 (60.3%)	89 (66.9%)	1.00	Reference	0.13	236.0	248.5
		*CT*	29 (39.7%)	44 (33.1%)	1.64	(0.86–3.16)			
***ABCC5*** **(rs9838667)**									
Codominant		*AA*	47 (64%)	88 (66%)	1.00	Reference	0.41	235.8	251.4
		*AC*	15 (21%)	37 (28%)	2.61	(1.05–6.48)			
		*CC*	11 (15%)	8 (6.0%)	1.10	(0.46–2.62)			
Overdominant		*AA-CC*	44 (60.3%)	96 (72%)	1.00	Reference	0.11	269.8	286.5
		*AC*	29 (39.7%)	37 (28%)	1.29	(0.70–2.35)			
***ABCC5*** **(rs3792585)**									
Codominant		*TT*	35 (48%)	93 (69.9%)	1.00	Reference	0.003	250.9	270.8
		*TC*	20 (27.4%)	36 (27.1%)	1.42	(0.72–2.81)			
		*CC*	18 (24.7%)	4 (3%)	11.2	(3.50–35.91)			
Recessive		*TT-TC*	55 (75.3%)	129 (97%)	1.00	Reference	0.004	249.9	266.5
		*CC*	18 (24.7%)	4 (3%)	9.99	(3.19–31.28)			

AIC, Akaike Information Criterion; BIC, Bayesian Information Criterion.

* p < 0.05.

The associations among polymorphisms and ALL were determined by the OR test (CI 95%, *p* < 0.05). The results shown in Table 3 emphasize that the *CT* carriers for *COL18A1* rs2274808 have a significant risk for ALL (OR = 2.55; CI95%, 1.11–5.83). This result was also found in the dominant model *CT-TT* in relation to the wild type genotype. For *SLC19A1* rs2838956, the best model was an over-dominant strategy where *AG* subjects showed an atypical association with values of OR = 44.69.

Moreover, *ABCB1* rs1045642 increased the ALL risk for subjects carrying the *TT* genotype in both the codominant and recessive models. The *ABCB1* rs1128503 did not result in a risk association to ALL.

The AC genotype for *ABCC5* rs9838667 SNP was associated with ALL susceptibility (OR = 2.61, CI 95% 1.05–6.48). However, the *ABCC5* rs3792585 SNP showed an increased risk for ALL in subjects carrying the CC genotype, which was the same result found in the recessive model.

Table 4 shows significant paired combinations for the closest SNPs in each gene and their association with ALL susceptibility. The results indicated that the *COL18A1* (rs2274808)+*SLC19A1* (rs2838956) combination, in relation to the wild type genotype *(CC*+*AA)*, had a significant association to ALL among the *CC*+*AG, CT*+*AG, TT*+*AG*, and *TT*+*GG* combinations. The combination of *ABCB1 SNPs* (rs1045642+rs1128503), particularly the combinations of *TT*+*CT* and *TT*+*TT*, presented a significant association to ALL. Finally, the total homozygote combination of *CC*+*CC* showed an increased risk association with ALL (OR: 5.33, CI 95% 2.59–1097). In contrast, the *AC*+*TT* and *AC*+*TC* combinations were shown to be protective against ALL.

**Table 4:** **Combined risk analyses for SNPs in relation to ALL**.

**Combined SNPs**	**Case**	**Control**	**OR**	**95%CI**	*p*-value^*^
***COL18A1*** **(rs2274808)**+***SLC19A1*****(rs2838956)**					
*CC+AA*	39	155	1.00	–	–
*CC+AG*	110	167	2.62	(1.71–4.01)	< 0.0001
*CT+AG*	86	83	4.12	(2.59–6.54)	< 0.0001
*TT+AG*	90	66	5.42	(3.38–8.70)	< 0.0001
*TT+GG*	21	31	2.69	(1.39–5.19)	0.003
***ABCB1*** **(rs1045642**+**rs1128503)**					
*CC+CC*	70	146	1.00	–	–
*TT+CT*	44	56	1.64	(1.01–2.66)	0.040
*TT+TT*	24	25	2.00	(1.06–3.75)	0.029
***ABCC5*** **(rs9838667**+**rs3792585)**					
*AA+TT*	82	181	1.00	–	–
*AC+TT*	50	181	0.61	(0.40–0.91)	0.017
*AC+TC*	35	124	0.62	(0.39–0.98)	0.041
*CC+CC*	29	12	5.33	(2.59–10.97)	< 0.0001

* Only significant genotype combinations are included (p < 0.05).

The artificial neural network analysis determined that the best general model was that of one layer with 48 hidden neurons, an error-assay of 11.7% and an error-training of 14.8%, respectively. The results indicated the most important normalized variables and pondered percentages for a response to ALL were *ABCB1* rs1045642 (72.8%), *COL18A1* rs2274808 (56.7%), and *ABCC5* rs3792585 (40.9%), resulting in the following regression expression:

$$\begin{array}{l} \text{ALL susceptibility}=4.201-0.921^{*}\text{ rs}2274808-1.647\\ \;{\;}^{*}\text{ rs}1045642-1.066{\;}^{*}\text{ ​​ABCC}5\text{ rs}3792585. \end{array}$$

## Discussion

Pharmacokinetics, that is, absorption, distribution, metabolism, and excretion (ADME) describe the disposition of a xenobiotic within an organism. The four processes all influence the drug exposure to the tissues and hence, influences efficacy and safety of a compound/drug. In this sense, as in many other compounds, ADME process for folates requires membrane transporters (e.g., RFC1, ABC; Lage, 2008; Wolking et al., 2015). Due to its role on nucleotide metabolism and DNA synthesis, polymorphisms in genes associated to folate pathways may have influence in cancer susceptibility and chemotherapeutic response to methotrexate, an antifolate-antineoplastic drug (Ross and Doyle, 2004; Steinberg et al., 2007; Galbiatti et al., 2013). It has been shown that SNPs in folate-associated pathways give rise to different phenotypes with direct clinical implicances and/or pathological specific reactions (O'leary et al., 2006).

In order to evaluate this, in this study we analyzed six polymorphisms in genes *RFC1* and *ABC's* and also *COL18A1* (influencing the activity of these transporters), in relation to the risk of occurrence of ALL.

Our results suggest that *COL18A1* rs2274808 could represent a risk factor for ALL. In addition, the dominant model, which groups these 2 subpopulations, suggests a similar behavior. The activity of *COL18A1* rs2274808 was further validated by a study which determined that a defect in *COL18A1* would change endostatin synthesis in a Salvadorian population (Mahajan et al., 2010), consequently leading to an antiangiogenic disorder, such as Knoblock syndrome and ALL; however, some authors have indicated that children with ALL have variable levels of endostatin (Dagdas et al., 2011), which makes it difficult to accurately explain its relationship with the disease (Schneider et al., 2007).

*SLC19A1* rs2838956 was associated with the occurrence of ALL (dominant model *AG*), which is similar to the results reported by De Jonge et al. (2009) who found that a 80 G>A SNP was significantly associated with ALL for both the heterozygote and homozygote genotypes (Table 3). In contrast to our findings, Yeoh et al. (2010) found in their case-control study of Malaysian and Chinese populations (321 and 346 individuals, respectively) that ALL children carrying the *G*>*A* genotype had protection against the disease. However, the analysis of binary combinations showed that 4 SNPs are associated with a higher tendency to develop ALL, which is a new finding.

The scientific literature, as reported by Ma et al. (2015a,b) in two meta-analysis indicate no clear association between this polymorphism and risk to ALL. In our study, we found that the rs1045642 ABCB1 SNP (known as 3435C > T) was associated with a risk of ALL occurrence. This finding is in agreement with the studies by Jamroziak et al. (2004) in Polish children carrying the *TT* genotype and by Qian et al. (2012) in a pediatric Chinese population at risk for leukemogenesis in *CT-TT* vs. *CC* individuals. However, the study by Hua-Jie et al. showed no associations with the disease in a Chinese population (Hua-Jie et al., 2011). Moreover, the results of Drain et al. (2009) suggested that individuals carrying the *ABCB1* rs1045642 variation should have a beneficial impact because the overall survival rate would be extended. In our risk analysis for *ABCB1* rs11288503, we found no relationship between this SNP and ALL susceptibility, which contrasts the report by Ma et al. (2015a). However, when we performed the study on the combination of both SNPs (*ABCB1* rs1045642+rs1128503), we observed that the rare allele combinations (*TT*+*CT* and *TT*+*TT*) were associated with a higher risk for ALL, which explains why children carrying such alleles are more likely to acquire the disease than those who have combinations with wild type genotypes. This result is similar to the reported by Semsei et al. (2008).

The results for the analyzed SNPs of *ABCC5* (rs9838667 and rs3792585) demonstrated that only rs3792585 showed a significant association with ALL susceptibility for subjects carrying the *CC* genotype (Table 2). However, when both SNPs were combined only homozygote genotype combinations showed an increased risk of leukemogenesis (Table 4). Finally, the *AC*+*TT* and *AC*+*A/T* combinations of *ABCC5* were observed as protection factors for ALL (Table 4).

For the risk analyses we used inheritance models to determine risk genotypes for ALL, which is based in the idea that the rare allele modify the risk, therefore the codominant model, used as reference, explains a different risk for each genotype which are non-additive. In the dominant model the risk for heterozygote genotype is similar to the homozygote for the rare allele. Conversely, in the recessive model the wild type genotype and heterozygote genotype have similar risk. On the other hand, in the over-dominant model the wild type genotype and the homozygote for rare allele have similar risk. Finally, In the additive model the basic idea is that a copy of the allele produces half of the risk of the two alleles (Iniesta et al., 2005; Zintzaras and Lau, 2008). In this respect, we choose the most probable model (besides the codominant) for each polymorphic variants to study risk. In this sense, our results (Table 3) showed that for *COL18A1* (rs2274808) the best model was de codominant, for *SLC19A1* (rs2838956) was the over-dominant model, for ABCB1 (rs1045642) was the codominant, non significantly different to risk obtained from the recessive model, for the *ABCB1* (rs1128503) and *ABCC5* (rs9838667) there were not significant associations for both the codominant or over-dominant models. Finally for the *ABCC5* (rs3792585) both the codominant and recessive models gave a significant risk to ALL.

One limitation of this study was the modest sample size of the cases (73). However, in our country, studies using children are quite restricted, even more whether they are patients. Moreover, considering there is an obligation to get both an informed consent and an informed assent, we believe this is a good starting number of subjects. In relation to that, there are several recent published studies with relatively small number of children (Roy Moulik et al., 2015; Amitai et al., 2016) and even more, with adults with ALL (Hareedy et al., 2015). Despite this, we truly believe our results are only a preliminary contribution regarding the ALL susceptibility of our Mexican pediatric population.

In summary, we found that 4 SNPs (*COL18A1* rs2274808, *SLC19A1* rs2838956, *ABCB1* rs1045642, and *ABCC5* rs3792585) either alone, or in some combinations, were associated with a higher risk for ALL in Mexican children. In contrast, children carrying the *AC*+*TT* or *AC*+*TC* combined genotypes of *ABCC5* seemed to be protected against ALL. These results suggest that the inter-individual variability of each patient in genes associated with the folate transport pathway influences the development of ALL.

## Author contributions

FZ, Analysis, interpretation of data, design and drafting the work, final approval of the version to be published. IL, conception and design of the work, interpretation of data, critical review of the content, financial support, final approval of the version to be published. VL, Interpretation of data, critical review of the content, final approval of the version to be published. AL, Interpretation of data, critical review of the content, final approval of the version to be published. AR, Interpretation of data, critical review of the content, final approval of the version to be published. MS, Interpretation of data, critical review of the content, final approval of the version to be published. CG, Interpretation of data, critical review of the content, final approval of the version to be published. MA, Interpretation of data, critical review of the content, final approval of the version to be published. MR, Interpretation of data, critical review of the content, final approval of the version to be published. LQ, Design of the work, interpretation of data, critical review of the content, financial support, final approval of the version to be published.

## Funding

This work was partly financed with Chilean Fondecyt Grant no. 1140434 and the National Polytechnic Institute-CIIDIR, Durango, Mexico.

### Conflict of interest statement

The authors declare that the research was conducted in the absence of any commercial or financial relationships that could be construed as a potential conflict of interest.
